# Prolapse of duodenal bulb from esophageal hiatal hernia: A case report

**DOI:** 10.1016/j.ijscr.2023.109014

**Published:** 2023-11-04

**Authors:** Wako Inoue, Shoichiro Mukai, Yasufumi Saito, Toshikatsu Fukuda, Hideki Ohdan

**Affiliations:** a Department of Surgery, ChugokuRosai Hospital, 1-5-1, Hirotagaya, Kure City, Hiroshima 737-0193, Japan; b Department of Gastroenterological and Transplant Surgery, Graduate School of Biomedical and Health Sciences, Hiroshima University, 1-2-3 Kasumi, Minami-ku, Hiroshima 734-8551, Japan

**Keywords:** Esophageal hiatal hernia, Duodenal bulb, Case report

## Abstract

**Introduction:**

The number of patients with hiatal hernia has increased. Paraesophageal and mixed hiatal hernias are absolute indications for surgical treatment due to the possibility of blood flow disturbances to the stomach and other organs.

**Case presentation:**

A 77-year-old woman with a history of type IV esophageal hiatal hernia (under observation), multiple operations presented with a chief complaint of vomiting. She was diagnosed with a type IV esophageal hiatal hernia with incarceration of the duodenal bulb into the mediastinum. Although the incarceration was relieved with conservative treatment, the patient was at a high risk for recurrence; therefore, surgical hernia repair was performed.

Intraoperatively, the hernia portal was severely dilated and the duodenal bulb was easily accessible to the mediastinum due to its high mobility. Fundoplication was performed using the Toupet procedure. No stenosis at the fundoplication site was observed on intraoperative upper gastrointestinal endoscopy.

**Discussion:**

The causes of prolapse and incarceration of the duodenal bulb into the mediastinum were speculated to be weakening of the tissue due to aging, adhesion of the omentum to the hernia portal due to chronic prolapse of the stomach toward the mediastinum, increased intra-abdominal pressure due to a rounded back, and anatomical shortening of the distance between the esophageal hiatus and the duodenal bulb. The Toupet method was used as it is associated with a lower incidence of dysphagia.

**Conclusion:**

Further investigation is needed to determine the best surgical technique.

## Highlights

• Esophageal hiatal hernia is anatomically classified into four types.
• Few reports of duodenal bulb prolapse have been made.
• The patient had no history of bowel malrotation or Kocher's maneuver.
• The indications for the surgical treatment of esophageal hiatal hernias remain unclear.
• The Toupet method was used as it is associated with a lower incidence of dysphagia.

## 1 Introduction

The number of patients with hiatal hernia has increased among the elderly population. Around 60 % of people over the age of 50 have hiatal hernia. Hiatal hernia is caused by congential anomaly, acquired vulnerability of connective tissue due to obesity or aging, increased abdominal pressure. Types II-IV together account for 5–15 % of all hiatal hernias. Paraesophageal and mixed hiatal hernias must be treated surgically because of the potential for impaired blood flow to organs. This work is reported according to the SCARE criteria [1].

## 2 Presentation of case

**Fig. 1:**
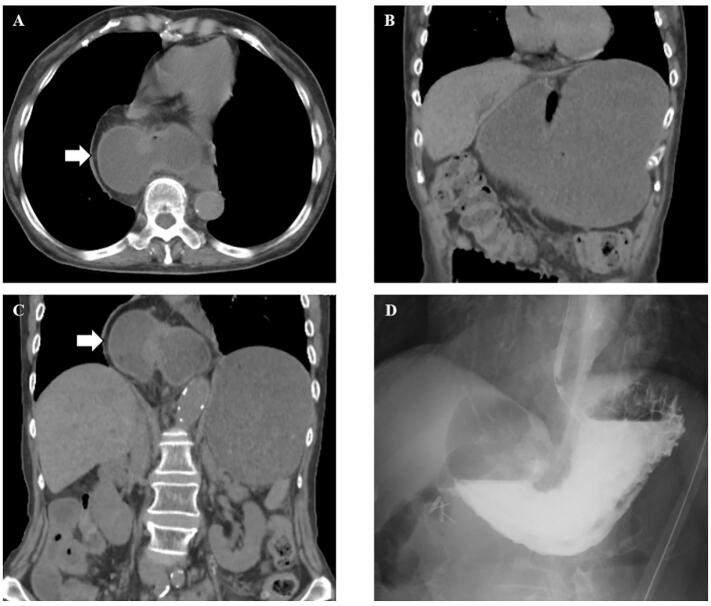
A, B, C: Duodenal prolapse and gastric dilatation within the mediastinum are observed on computed tomography (arrow indicates the duodenum bulb within the mediastinum). D: An enema shows improvement in the duodenal prolapse and gastric dilatation after decompression using a nasogastric tube.

A 77-year-old woman with a history of laparoscopic cholecystectomy for acute cholecystitis, total hysterectomy for cervical cancer, five laparotomies for adhesive intestinal obstruction, and type IV esophageal hiatal hernia presented with a chief complaint of vomiting after supper the same day. She vomited three times and vomited all her supper. The patient was under observation for the type IV esophageal hiatal hernia as she did not desire surgery. Blood tests showed no abnormal findings apart from mild dehydration. Computed Tomography (CT) revealed gastric dilation and prolapse of the pylorus to the duodenal bulb into the mediastinum. Contrast-enhanced CT was not performed due to age and dehydration. The patient was diagnosed with esophageal hiatal hernia incarceration (Fig. 1A, B, C). Conservative treatment including a nasogastric tube, fasting resolved the patient's symptoms. An oral contrast examination showed that the duodenal bulb no longer incarcerated, though the mobility of the pyloric region and duodenal bulb was severe (Fig. 1D). The patient was at high risk for incarceration recurrence. Therefore, a laparoscopic esophageal hiatal hernia repair was performed.

**Fig. 2:**
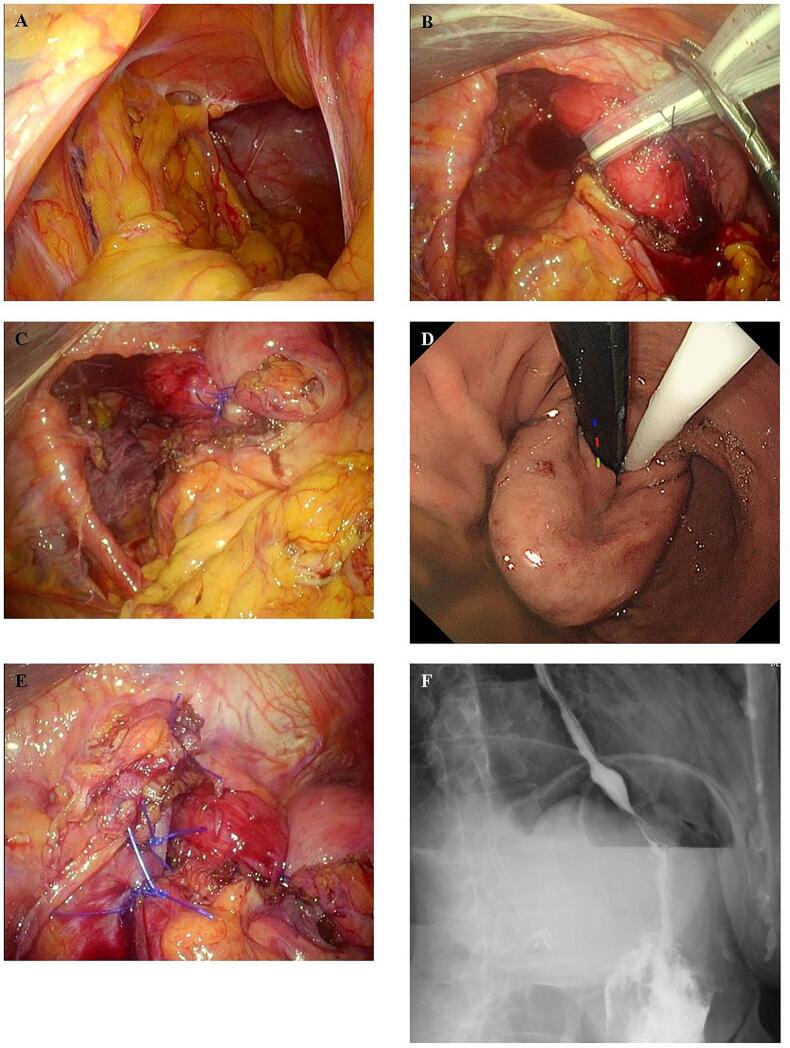
A: Dilation of the hernial portal and adhesion of the omentum within the hernial sac are shown intraoperatively. B: The esophagus is taped, and approximately 4 cm is retracted into the abdominal cavity. C: Fundoplication is performed using the Toupet procedure. D: Intraoperative gastrointestinal endoscopy confirms that there is no stenosis at the fundoplication site and that the scope can be passed through. E: The right and left diaphragmatic crus are ligated for esophageal hiatus formation. F: A postoperative radiographic contrast study reveals no evidence of stenosis or reflux.

Despite multiple surgeries, few adhesions were noted in the upper abdomen; however, a severely dilated hernia portal was observed. Although the duodenal bulb was no longer incarcerated, it had high mobility (Fig. 2A). The gastrosplenic ligament and the adhesions around the abdominal esophagus, the greater and lesser curvature sides of the cardiac part, and the left and right diaphragmatic crus were dissected. The esophagogastric junction was confirmed to be able to be retracted into the abdominal cavity by approximately 4 cm (Fig. 2B). Fundoplication was performed using the Toupet procedure. Intraoperative upper gastrointestinal endoscopy confirmed that there was no stenosis at the fundoplication site (Fig. 2C and D). The right and left diaphragmatic crus were partially sutured, and size of the esophageal hiatus was reduced (Fig. 2E). A postoperative radiographic contrast study showed no evidence of stenosis or reflux (Fig. 2F). The patient was discharged from the hospital 14 days postoperatively, with no recurrence to date.

## 3 Discussion

Esophageal hiatal hernia is a condition in which an intra-abdominal organ prolapses into the mediastinum via the esophageal hiatus in the diaphragm. It is anatomically classified into four types: type I, sliding hernia; type II, paraesophageal hernia; type III, mixed hernia with a sliding hernia and paraesophageal hernia; and type IV, combined hernia in which the stomach, omentum, prolapse into the mediastinum [2]. Type I is the most common type of esophageal hiatal hernia, accounting for up to nearly 95 % of cases, whereas types II-IV account for 5–15 % of cases [2]. Surgery is indicated in patients with gastric evacuation disorders, severe gastroesophageal reflux, anemia, respiratory symptoms such as shortness of breath, or strangulation caused by gastric torsion [3]. Few studies regarding duodenal bulb prolapse, as in the patient in this report, have been reported.

Compared to Type I patients, Type II-IV patients tended to have a mean age of 9 years older, more symptoms, higher BMI, larger hernia defects, higher ASA scores, and at least one underlying disease. Against this background, the incidence of postoperative complications was higher than in Type I patients. In addition, the size of the hernia portal and the presence of prolapsed organs may require more complex procedures and increase reoperations related to intraoperative organ damage and postoperative complications. Laparoscopic surgery for type II-IV hiatal hernias should be performed in centers with experienced surgeons. Those with obstruction or ischemia are indications for emergency surgery, with an 18 % chance of developing such acute symptoms in patients under follow-up and a 5.4 % mortality rate from emergency surgery. The lifetime risk of death from a hiatal hernia of the esophagus in patients under follow-up is about 1 %, suggesting that one in five patients aged 65 years is worth undergoing hernia repair to prevent complications [4].

Generally, the duodenum is anatomically fixed to the retroperitoneum and has little mobility. In this patient, there was no history of bowel malrotation or Kocher's maneuver; however, the fixation of the duodenal bulb to the retroperitoneum was very loose, increasing the mobility of the duodenal bulb. The causes of prolapse and incarceration of the duodenal bulb into the mediastinum were speculated to be weakening of the tissue with aging, adhesion of the omentum to the hernia portal due to chronic prolapse of the stomach toward the mediastinum (causing the duodenal bulb to be pulled toward the mediastinum for a long time and to gradually lose its attachment to the retroperitoneum), increased intra-abdominal pressure due to a rounded back, and anatomical shortening of the distance between the esophageal hiatus and the duodenal bulb.

Medical therapies are the preferred treatment for esophageal hiatal hernias. However, according to the guidelines of the Society of American Gastrointestinal Endoscopic Surgeons, surgical treatment is indicated in patients in whom medical treatment is not effective and surgical treatment is desirable due to various factors (such as age, duration of treatment, and medical costs). Surgical treatment is also preferred in patients with Barrett's esophagus, stenosis, severe esophagitis, asthma, hoarseness, cough, chest pain, aspiration symptoms, severe reflux (based on 24-hour pH monitoring), or bleeding or dysphagia due to a large esophageal hiatal hernia [5].

Surgical procedures for esophageal hiatal hernia include the Allison [6] and Belsey-Mark IV [7] transthoracic methods and the Nissen [8], Toupet [9], and Hill [10] transabdominal methods. In recent years, laparoscopic surgery has been used instead of conventional open surgery, as both methods have comparable results [11,12]. Paul et al. reported that mesh reinforcement reduces the recurrence rate by approximately 24% [13,14]. Similarly, a meta-analysis by Tam et al. found a significant reduction in the recurrence rate with the use of mesh though the reoperation rate was not significantly reduced [15]. There are no difference in postoperative recurrence or reoperation rates with or without the use of mesh and no established guidelines, and the indications for its use are controversial [16].

We use mesh when the hernia portal is large and the tissue is fragile; however, in this patient, the hernia portal was not large enough to require a mesh. The Toupet technique is used for fundoplication; a previous study reported that postoperative dysphagia is more likely to occur with the Nissen technique than with the Toupet technique [17]. In contrast, other studies reported that there is no difference between the two techniques at one year or more postoperatively [17,18]. Several studies indicate that the anti-reflux effect is not different between the two techniques [19]. We use the Toupet method because the incidence of dysphagia is low. In this patient, the esophageal hiatus was sutured to avoid stricture and the gastric fundus and diaphragm were secured to prevent reprolapse.

## 4 Conclusion

Although the patient had been treated conservatively and the hernia was reduced, surgery was performed due to the high possibility of recurrence of the hernia. Preoperative tests confirmed that the adhesions in the upper abdomen were mild, and the patient underwent surgery laparoscopically.

The patient underwent surgery using the Toupet technique to form a fundoplication and did not develop dysphagia. Further investigation is needed to determine the best surgical technique for esophageal hiatal hernia.

## Provenance and peer review

Not commissioned, externally peer-reviewed.

## Ethical approval

A case report is exempt from ethical approval in our institution.

## Funding

There are no sources of funding to declare.

## Consent

Written informed consent was obtained from the patient for publication of this case report and accompanying images. A copy of the written consent is available for review by the Editor-in-Chief of this journal on request.

## Author contribution

WI drafted the article. SM and TF had revised the manuscript critically. YS was 1st assistant of operation. HO was supervision of this manuscript. All authors contributed to study concept or design at this submission and approved the final version.

## Guarantor

Dr. Shoichiro Mukai.

## Registration of research studies

Not applicable.

This is a case report.

## Conflict of interest statement

No financial/personal relationships/conflicts of interest.
